# Nonsteroidal anti-inflammatory drug use is associated with improved activities of daily living and rehabilitation in older adult patients following a fracture: a retrospective cohort study

**DOI:** 10.1186/s40780-025-00445-2

**Published:** 2025-05-07

**Authors:** Eiji Kose, Hidetatsu Endo, Hiroko Hori, Shingo Hosono, Chiaki Kawamura, Yuta Kodama, Takashi Yamazaki, Toshimi Kimura

**Affiliations:** 1 Department of Pharmacy, Juntendo University Hospital, 3-1-3 Hongo, Bunkyo-ku, Tokyo, 113-8431 Japan; 2 Department of Pharmacy, Ogaki Tokushukai Hospital, 6-85-1 Hayashi-chou, Ogaki, Gifu, 503- 0015 Japan; 3 Faculty of Pharmacy, Juntendo University, 6-8-1 Hinode, Urayasu City, 279-0013 Chiba Japan

**Keywords:** Activities of daily living, Older adult patients, Functional independence measure-total gain, Nonsteroidal anti-inflammatory drugs

## Abstract

**Background:**

Insufficient rehabilitation due to postfracture pain can result in muscle atrophy and joint contractures, which may affect the improvement of activities of daily living (ADL). This study investigated the impact of using nonsteroidal anti-inflammatory drugs (NSAIDs) on the improvement of ADL in older adult patients with fractures admitted to a convalescent rehabilitation unit.

**Methods:**

Of 489 older adult patients with fractures from January 2017 to June 2019, 261 fulfilled the requirements for this retrospective cohort analysis. Patients who had convalescent rehabilitation following a fracture were categorized into two groups: those who used NSAIDs and those who did not. The functional independence measure-total gain (FIM-total) score, which was used for evaluating ADL, was the main outcome measure. We ascertained the independent relationship between NSAIDs use and rehabilitation outcomes using a multiple linear regression analysis. Covariates selected to correct bias included age, sex (male), BMI, hypertension, dementia, cardiovascular disease, cerebrovascular disease, upper limb paralysis, femoral fracture, lumbar compression fracture, thoracic compression fracture, pelvic fracture, patellar fracture, FIM-total at admission, number of drugs, acetaminophen use.

**Results:**

The mean participant age was 82.3 ± 7.4 years, 69 (26.4%) of them were men, and 94 (36%) used NSAIDs. Multiple linear regression analysis revealed that NSAIDs use was independently associated with FIM-total gain during hospitalization (β=2.311, P=0.013).

**Conclusions:**

These findings suggest that the appropriate use of NSAIDs may play a beneficial role in maximizing rehabilitation outcomes. However, careful monitoring for potential adverse effects is essential, particularly in older adults.

## Background

Implementing appropriate rehabilitation for patients with fractures is crucial. In the case of a fall or accident resulting in a fracture, the selected course of treatment, whether it involves surgical intervention or immobilization with a cast, is contingent upon the location and type of the fractured bone. Owing to the prolonged immobilization of surgical and fracture sites, a risk of muscle atrophy and joint contractures exists. Additionally, surgical procedures or fractures may induce tissue damage or pathological changes, thereby leading to joint pain. Therefore, postfracture rehabilitation aims to minimize the reduction in muscle strength and the occurrence of joint contractures during the period of surgery or immobilization. Therefore, to promote fracture healing, we perform exercises, including strength training for muscles outside the fractured area, joint range of motion exercises, and activities such as walking and stair climbing, ensuring they do not hinder fractured bone recovery. Furthermore, in cases where fractures result from falls, aging and osteoporosis are considered potential causes. Therefore, rehabilitation focusing on preventing subsequent falls and fractures is crucial.

Effective pharmacological pain management is essential for individuals with fractures. Insufficient rehabilitation due to postfracture pain can result in muscle atrophy and joint contractures. Doctors occasionally use acetaminophen to treat postfracture pain. Although acetaminophen exhibits antipyretic and analgesic effects, its anti-inflammatory properties are relatively weak [1]. This finding suggests that although increasing acetaminophen dosage can alleviate moderate pain, individuals frequently perceive its overall effectiveness as limited [2]. Clinical practice frequently favors nonsteroidal anti-inflammatory drugs (NSAIDs) [3]. NSAIDs, known for their analgesic and anti-inflammatory effects, are among the most commonly prescribed medications, as evidenced by their inclusion in the World Health Organization’s Essential Medicines Model List [4]. When NSAIDs use alleviates postfracture pain, the potential to avoid interruptions in rehabilitation caused by pain is observed, thereby maximizing the effectiveness of the rehabilitation.

To the best of our knowledge, studies examining whether NSAIDs use in the rehabilitation setting affects the improvement of activities of daily living (ADL) in patients with fractures remain scarce. There are reports that the application of rehabilitation nutrition [5] in older patients with hip fractures has led to significant improvements in their ADL [6]. To date, there have only been studies of deprescribing medications being associated with improved ADL through rehabilitation [7, 8, 9], and no studies on patients who are older and have undergone a fracture have been conducted. Showing a correlation between NSAIDs use and ADL improvement through rehabilitation would contribute to the seamless implementation of appropriate rehabilitation for patients with fractures.

This study investigated the impact of NSAIDs administered for fracture-related pain management on improvements in ADL through rehabilitation in older patients following fractures.

## Materials and methods

### Study’s context, participants, and design

This retrospective cohort analysis was conducted in the convalescent rehabilitation units of a 283-bed acute care hospital. This study spanned from January 2017 to June 2019. Newly admitted patients to the convalescent rehabilitation units had to be at least 65 years old. The following were the exclusion criteria: missing data and refusal to take part in the study; presence of a primary disease except for bone fracture (stroke, hospital-associated deconditioning, and others); and relocation to a different medical facility or unit as a result of changes in health status during the rehabilitation process.

### Data collection and evaluation

On admission, we recorded baseline characteristics, including age, sex, comorbid conditions, paralysis, length of hospital stay, family support, and medication data. Furthermore, upon admission, data on albumin and C-reactive protein levels in the blood were collected. Nutritional risk was assessed using body mass index (BMI) calculation. A multidisciplinary team including occupational therapists, speech–language pathologists, physical therapists, and nurses with long and extensive clinical experience calculated the functional independence measure (FIM) score as the total of physical and cognitive functions (FIM-total) [10]. All participants received suitable rehabilitation according to their clinical judgment, irrespective of their FIM score or length of hospital stay.

### Outcome measures

The total gain of FIM was the main outcome measure. We computed the FIM-total gain by subtracting the FIM-total at admission from the FIM-total at discharge. Thirteen items comprised the FIM’s motor domain (FIM-motor), whereas five items comprised the FIM’s cognitive domain (FIM-cognitive). We evaluated the movements using a 7-point ordinal scale, which ranged from completely dependent to completely independent. The FIM-motor, FIM-cognitive, and total FIM scores varied from 13 to 91, 5 to 35, and 18 to 126 points, respectively. Lower scores suggested more reliance. Length of stay was the secondary outcome.

### Sample size calculation

We determined the sample size using data from a previous study [11], which revealed a normally distributed FIM-total score with a standard deviation (SD) of 22.69 for patients admitted to the hospital. To show that our results are valid, we would need a sample size of at least 90 individuals in each group, with a real mean value difference of 11 between the groups (NSAIDs use at admission and non-NSAIDs use). This requirement would indicate that the null hypothesis would be rejected with a power of 0.9 and an alpha error of 0.05.

### Statistical analysis

We presented categorical data as numbers (%), nonparametric data as medians and 25th − 75th percentiles (interquartile range), and parametric data as means and standard deviation (SD). Bivariate analysis was divided into NSAIDs use and non-NSAIDs use groups on the basis of whether NSAIDs were used at admission. We used t-tests, Mann–Whitney U tests, and chi-square tests for between-group comparisons depending on the type of variable data. Multiple linear regression models were used to investigate the independent relationship between NSAIDs use and FIM-total gain and length of hospital stay. Covariates selected to correct bias included age, sex (male), BMI, hypertension, dementia, cardiovascular disease, cerebrovascular disease, upper limb paralysis, femoral fracture, lumbar compression fracture, thoracic compression fracture, pelvic fracture, patellar fracture, FIM-total at admission, number of drugs, acetaminophen use. We adjusted bias for common confounders through a sequence of multivariate analyses. We measured multicollinearity using the variance inflation factor (VIF) and considered a VIF value of < 3 as evidence of nonmulticollinearity. All statistical analyses were performed using JMP Pro (version 13; SAS Institute, Cary, NC, USA). *P* < 0.05 was considered statistically significant.

## Results

**Fig. 1:**
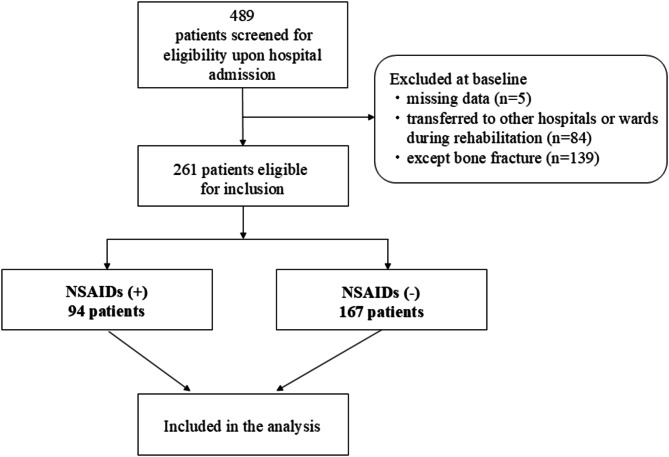
Flowchart of participant screening, inclusion criteria, and follow-up

Of the 489 older adult patients admitted during the study period, 5 had missing data, 84 were transferred to different hospitals or wards for rehabilitation, and 139 had other primary diseases excluding fractures (cerebral hemorrhage, 21; cerebral infarction, 49; subarachnoid hemorrhage, 16; hospital-associated deconditioning, 51; and others, 2). Ultimately, this study included 261 patients (Fig. 1). The baseline characteristics of the participants are summarized in Table 1. Of them, 69 (26.4%) were male, and the mean age was 82.3 (SD, 7.4) years. Most patients at baseline exhibited physical reliance, as observed by the median FIM-total score of 74 (range, 59–89.5). The median length of hospital stay was 39 (range, 27–53.5) days. Between-group analysis showed that the NSAIDs use group had much greater number of total drugs at baseline, BMI, and FIM-cognitive scores than the non-NSAIDs use group.

**Table 1:** Baseline characteristics of participants and comparison between groups of patients with and without NSAIDs use

	Total (*N* = 261)	NSAIDs use Group (*N* = 94)	Non-NSAIDs use Group (*N* = 167)	*P* value
Age, *y*	82.3 (7.4)	81 (7)	83.1 (7.5)	0.032
Sex, *male n*, (%)	69 (26.4)	21 (22.3)	48 (28.7)	0.256
BMI, *kg/m*^*2*^	20.6 [18.2, 23]	21 [19, 24.1]	20.1 [17.7, 22.6]	0.020
Comorbid conditions *n*, (%)				
Cardiac disease	41 (15.7)	9 (9.5)	32 (19.2)	0.035
Hypertension	167 (64)	61 (64.9)	106 (63.5)	0.818
Dementia	28 (10.7)	11 (11.7)	17 (10.2)	0.704
Parkinson’s disease	5 (1.9)	2 (2.1)	3 (1.8)	0.851
Epilepsy	7 (2.7)	2 (2.1)	5 (3)	0.672
Cerebrovascular disease	36 (13.8)	13 (13.8)	23 (13.8)	0.990
Higher brain dysfunction	2 (0.8)	1 (1.1)	1 (0.6)	0.672
Paralysis *n*, (%)				
upper limbs	7 (2.7)	2 (2.1)	5 (3)	0.064
Fracture Sites *n*, (%)				
Femoral Lumbar Spine Thoracic Spine Pelvic Patellar Others	110 (42.1) 54 (20.7) 41 (15.8) 20 (7.7) 15 (5.7) 21 (8)	30 (31.9) 22 (23.4) 24 (25.5) 8 (8.5) 3 (3.2) 7 (7.5)	80 (47.9) 32 (19.2) 17 (10.2) 12 (7.2) 12 (7.2) 14 (8.4)	0.012 0.417 0.001 0.699 0.183 0.790
FIM, *score*				
-Total	74 [59, 89.5]	73 [59, 87.3]	75 [59, 90]	0.762
-Motor	47 [31, 58]	47 [30, 64.2]	48 [32, 58]	0.241
-Cognitive	29 [25, 35]	32 [25.5, 35]	28 [24, 35]	0.018
Laboratory data				
Alb, *g/dL*	3.5 (0.2)	3.5 (0.4)	3.5 (0.3)	0.889
CRP, *mg/dL*	0.3 [0.1, 1.1]	0.4 (0.1, 1.1)	0.3 (0.2, 1.2)	0.519
Family Support *n*, (%)	250 (95.8)	90 (95.7)	160 (95.8)	0.980
Medication data				
Number of total drugs	7 [4, 9]	7 [5, 9]	6 [4, 9]	0.029
Number of medication review	4 [2, 7]	4 [3, 7]	4 [2, 6]	0.096
Acetaminophen use *n*, (%)	94 (36)	22 (23.4)	72 (43.1)	0.002
Weak opioid use *n*, (%)	6 (2.3)	2 (2.1)	4 (2.4)	0.890

Alb, albumin; BMI, body mass index; CRP, C-reactive protein; FIM, Functional Independence Measure; NSAIDs, non-steroidal anti-inflammatory drugs

Data are expressed as means (standard deviation) for parametric data, while medians and 25th to 75th percentiles (interquartile range (IQR)) were used to describe nonparametric data, and numbers (%) were used to describe categorical data

A two-group analysis of the results for the NSAIDs and non-NSAIDs groups is presented in Table 2. Univariate analysis revealed significant differences between the NSAIDs and non-NSAIDs groups, with the FIM-total gain (36 [range, 24–51.3]) vs. 30 [19–41], *P* = 0.002, respectively). However, the length of hospital stay was not significant differences between two groups (35 [25–54] vs. 40 [29–53], *P* = 0.131, respectively). The results of the multivariate linear regression analysis are shown in Table 3. No variable multicollinearity was observed. NSAIDs use upon admission was independently correlated with FIM-total gain (β = 2.311, *P* = 0.013), but no significant correlated with length of stay (β = 0.081, *P* = 0.948).

**Table 2:** Univariate analyses of outcomes between NSAIDs use and non-NSAIDs use group

	Total (*N* = 261)	NSAIDs use Group (*N* = 94)	Non- NSAIDs use Group (*N* = 167)	*P* value
FIM-total gain, *score*	31 [20.5, 43.5]	36 [24, 51.3]	30 [19, 41]	0.002
Length of stay, *d*	39 [27, 53.5]	35 [25, 54]	40 [29, 53]	0.131

FIM, Functional Independence Measure; NSAIDs, non-steroidal anti-inflammatory drugs

**Table 3:** Multivariate analyses for patient outcomes of NSAIDs use

	FIM-total gain				Length of stay			
	β	B (95% CI)	VIF	*P* value	β	B (95% CI)	VIF	*P* value
Age	-0.262	-0.111 (-0.519, -0.006)	1.397	0.045	0.164	0.065 (-0.181, 0.508)	1.392	0.351
Sex (male)	-1.253	-0.063 (-3.152, 0.645)	1.100	0.195	0.037	0.002 (-2.522, 2.595)	1.100	0.977
BMI	0.348	0.072 (-0.129, 0.825)	1.155	0.152	-0.141	-0.027 (-0.783, 0.502)	1.154	0.667
Hypertension	0.284	0.016 (-1.535, 2.105)	1.173	0.758	-0.922	-0.048 (-3.371, 1.527)	1.170	0.459
Dementia	-6.450	-0.227 (-9.356, -3.544)	1.248	< 0.001	-1.324	-0.044 (-5.235, 2.587)	1.246	0.505
Cardiovascular disease	-1.652	-0.068 (-4.032, 0.762)	1.145	0.172	0.003	0.001 (-3.202, 3.208)	1.145	0.999
Cerebrovascular disease	1.087	0.042 (-1.534, 3.708)	1.208	0.415	-1.060	-0.039 (-4.588, 2.469)	1.207	0.555
Upper limb paralysis	-1.765	-0.028 (-8.041, 4.511)	1.182	0.580	-1.621	-0.024 (-10.074, 6.831)	1.182	0.706
Femoral Fracture	-0.245	-0.014 (-3.415, 2.924)	2.780	0.879	-2.108	-0.112 (-6.369, 2.154)	2.765	0.331
Lumbar Compression Fracture	0.151	0.001 (-3.371, 3.673)	2.232	0.933	-8.444	-0.374 (-13.064, -3.823)	2.063	0.001
Thoracic Compression Fracture	1.492	0.063 (-2.188, 5.173)	2.853	0.425	-8.661	-0.345 (-13.492, -3.828)	2.710	0.001
Pelvic Fracture	1.324	0.040 (-2.957, 5.606)	1.974	0.543	-6.556	-0.187 (-12.263, -0.850)	1.932	0.025
Patellar Fracture	-0.024	-0.001 (-4.479, 4.431)	1.716	0.992	-0.319	-0.008 (-6.320, 5.683)	1.716	0.917
FIM-total at admission	-0.564	-0.697 (-0.655, -0.473)	1.500	< 0.001	-0.191	-0.223 (-0.311, -0.071)	1.440	0.002
Number of drugs	0.201	0.041 (-0.323, 0.724)	1.347	0.451	0.194	0.037 (-0.511, 0.898)	1.345	0.589
Acetaminophen use	0.629	0.034 (-1.183, 2.441)	1.174	0.495	0.425	0.022 (-2.015, 2.866)	1.174	0.732
NSAIDs use	2.311	0.127 (0.501, 4.122)	1.184	0.013	0.081	0.004 (-2.385, 2.521)	1.184	0.948
Length of stay	-0.044	-0.046 (-0139, 0.051)	1.204	0.365				

BMI, body mass index; CI, confidence interval; FIM, Functional Independence Measure; NSAIDs, non-steroidal anti-inflammatory drugs; VIF, variance inflation factor

## Discussion

This study examined how NSAIDs use among older adult patients following a fracture is correlated with their ability to increase ADL during rehabilitation. The observation of a favorable relationship between NSAIDs use and ADL improvement during rehabilitation among older adult patients with fractures is the most significant result of this study. The lack of correlation between NSAIDs use and length of hospital stay is another notable observation. Few studies have suggested a beneficial effect of medication on functional recovery through rehabilitation compared with exercise or diet. Therefore, our findings may contribute to maximizing the efficiency of rehabilitation for older adults with fractures.

The NSAIDs used upon admission were positively and independently correlated with ADL improvement through rehabilitation. Essentially, NSAIDs use demonstrated a positive impact on functional recovery during rehabilitation. For individuals with fractures, rehabilitation and physical activities were also crucial parts of rehabilitation. With the significance of both nutritional and rehabilitation treatment in this group, “rehabilitation nutrition” appears to be beneficial for the recovery of patients with impairments [12]. In patients aged ≥ 65 years with proximal femoral fractures undergoing rehabilitation, enhanced nutritional therapy provided concurrently with early postoperative rehabilitation is recommended [6]. This approach aims to reduce mortality and complication rates, as well as improve ADL and muscle strength. Conversely, “rehabilitation pharmacotherapy” [13, 14] is considered valuable for understanding the relationship between medications and rehabilitation. Several reports have indicated that polypharmacy and potentially inappropriate medications may have a negative impact on the improvement in ADL and nutritional status through rehabilitation [15, 16, 17, 18, 19, 20, 21, 22, 23, 24]. Some studies have reported an association between deprescribing potentially inappropriate medications and psychotropic medications, as well as the improvement of ADL with rehabilitation [7, 8, 9]. However, to date, no other study has shown a direct correlation between medication use and the outcomes of interest, without deprescribing medications, and ADL improvement. Our results showed that NSAIDs are beneficial for patients undergoing rehabilitation following fractures and are considered clinically significant. NSAIDs alleviate postoperative and postfracture pain, thereby preventing pain-related interruptions in rehabilitation and maximizing rehabilitation effectiveness.

The use of NSAIDs to alleviate fracture pain is controversial owing to their potential adverse effects on bone repair. Animal research has shown this to be true, whereas clinical trials have shown conflicting outcomes. In 2019, a meta-analysis investigated how NSAIDs affected bone healing in long bone and spine fractures. Most of the studies were retrospective designs, which limit their practical applicability and emphasize the necessity for additional research in this area [25]. A recent meta-analysis reported that patients exposed to NSAIDs are more likely to experience delayed or nonunion in long bone and other fractures. However, short-term exposure to low doses did not show this impact [26]. Moreover, a retrospective comparison analysis of a single institution observed no statistically significant effects of ketorolac administered during the first 24 h following repair on healing time or incidence of nonunion for femoral or tibial shaft fractures [27]. Clinical trials failed to provide compelling evidence that NSAIDs impair bone repair. Union rates were unaffected by low-dose or short-duration exposure, suggesting that the effect is dose- or time-dependent. Therefore, one should avoid administering the drug more carelessly than necessary.

NSAIDs use upon hospital arrival did not significantly affect the length of hospital stay. Confounding variables selected during design, sample size calculations, and multivariate analysis adjustments likely affect the FIM-total gain, which is the major finding of this study. Therefore, the results of this study do not imply that NSAIDs use is ineffective in reducing the length of hospital stays. Numerous studies have previously reported factors that influence the length of hospital stays. Factors identified as influencing the length of hospital stay included the Timed Up and Go test, ADL on admission [28], nutritional status [29], poor mobility status [30], aging [30], BMI [31], female sex [31], and polypharmacy [32]. The use of NSAIDs for reducing postoperative and postfracture pain and maximizing rehabilitation effectiveness, potentially leading to improved ADL and, consequently, reduced length of hospital stay, is also considered. Therefore, the inclusion of previously reported confounding factors and the prioritization of reducing the length of hospital stay as the primary outcome require high-quality research.

Dementia was significantly negatively associated with improvements in ADL achieved through rehabilitation. Nevertheless, rehabilitation is critical not only for the maintenance of ADL, such as walking and toileting, but also for preserving muscle strength and joint range of motion. Furthermore, it plays a pivotal role in sustaining cognitive function and addressing psychiatric concerns, such as the prevention of depression, which are integral to the overall well-being of dementia patients. However, as the symptoms of dementia progress, the decline in cognitive functions such as comprehension and task execution abilities may hinder the acquisition of appropriate motor skills and movement patterns. Moreover, the behavioral and psychological symptoms of dementia, which often accompany the progression of the condition, can pose significant barriers to the effectiveness of rehabilitation interventions. Consistent with the findings of this study, previous reports have also identified the presence of dementia as a hindering factor for FIM gain [33, 34]. Given that the severity of dementia is believed to influence these hindrances, it is essential to implement individualized rehabilitation plans tailored to cognitive function, provide motivational support, and ensure the active involvement of family members and caregivers, along with appropriate environmental adjustments.

To enhance ADL and reduce the length of hospital stay for older adults with fractures, a holistic approach that addresses rehabilitation nutrition [5] and rehabilitation pharmacotherapy [13, 14] is required. The results of this study suggest that the effective use of NSAIDs for pain relief in postoperative and postfracture pain, combined with dietary and exercise programs, can maximize the effectiveness of rehabilitation. This finding indicates that the rehabilitation of older adults with fractures needs a multidisciplinary approach, pharmacotherapy, and nutrition. However, the administration of NSAIDs necessitates a thorough evaluation of renal function and meticulous consideration of concomitant medications including diuretics, angiotensin-converting enzyme inhibitors, and angiotensin II receptor blockers, particularly in light of the potential “triple whammy” effect.

This study had several limitations. First, it was a retrospective cohort study conducted in a single hospital in Japan; therefore, it did not demonstrate the generalizability. Second, when prescribing NSAIDs, gastrointestinal symptom, careful assessment of renal function and consideration of concomitant medications, particularly in the context of the triple whammy effect, are essential. However, due to the characteristics of the convalescent rehabilitation ward setting, frequent blood tests were not conducted, leading to insufficient data for a comprehensive analysis. Furthermore, we were unable to obtain sufficiently clear and comprehensive information from the available medical records regarding the incidence of gastrointestinal bleeding or concomitant medications that may affect renal function. Third, the length, kinds and dosage of NSAIDs use were not considered. Fourth, for sarcopenia, there was insufficient data to diagnose according to the Asian Working Group for Sarcopenia 2019 criteria [35], making a comprehensive analysis difficult. Fifth, data on rehabilitation interruptions due to pain were not considered. Finally, the severity of comorbidities was not considered. However, as no significant difference in ADL at admission was observed between the NSAIDs use and non-NSAIDs use groups, we do not believe that disease severity had a significant effect. Future prospective studies that take these factors into account are desirable for verification.

## Conclusions

NSAIDs use was independently associated with ADL improvement for older adults with fractures. From a rehabilitation pharmacotherapy perspective, older adults with fractures undergoing rehabilitation should use pharmacotherapy in addition to diet and exercise therapy to maximize the effectiveness of rehabilitation. However, the potential for drug-drug interactions leading to renal impairment and the adverse events associated with NSAID use have not been adequately studied and further rigorous evaluation is warranted.

## Data Availability

No datasets were generated or analysed during the current study.

## Abbreviations

ADL: activities of daily living
BMI: body mass index
FIM: functional independence measure
IQR: interquartile range
NSAIDs: nonsteroidal anti-inflammatory drugs
SD: standard deviation
VIF: variance inflation factor
