# Correction: Nonsteroidal anti-inflammatory drug use is associated with improved activities of daily living and rehabilitation in older adult patients following a fracture: a retrospective cohort study

**DOI:** 10.1186/s40780-025-00454-1

**Published:** 2025-05-30

**Authors:** Eiji Kose, Hidetatsu Endo, Hiroko Hori, Shingo Hosono, Chiaki Kawamura, Yuta Kodama, Takashi Yamazaki, Toshimi Kimura

**Affiliations:** 1 Department of Pharmacy, Juntendo University Hospital, 3-1-3 Hongo, Bunkyo-ku, Tokyo, 113-8431 Japan; 2 Department of Pharmacy, Ogaki Tokushukai Hospital, 6-85-1 Hayashi-chou, Ogaki, Gifu, 503- 0015 Japan; 3 Faculty of Pharmacy, Juntendo University, 6-8-1 Hinode, Urayasu City, 279-0013 Chiba Japan

**Correction to: Journal of Pharmaceutical Health Care and Sciences (2025) 11:39**

10.1186/s40780-025-00445-2

Following publication of the original article [1], the authors reported that the second author’s name, “Hidetatsu Endo”, is listed twice on the author list.

The correct author list has been provided in this Correction and the original article [1] has been corrected.
